# Temporal Probability-Guided Shifts in Temporal Preparation Away from the Beat Under a Distracting Rhythm in Aging

**DOI:** 10.3390/bs16030453

**Published:** 2026-03-19

**Authors:** Zhihan Xu, Siyu Chen, Zhili Han, Yuqing Jiang, Ting Guo, Sa Lu

**Affiliations:** 1 Research Center of Language and Cognition, Ningbo University of Technology, Ningbo 315211, China; xzhan22@163.com (Z.X.); chensiyu_1020@163.com (S.C.); jiangyuqing0120@163.com (Y.J.); 2 School of International Studies, NingboTech University, Ningbo 315100, China; hanzhili@nbt.edu.cn

**Keywords:** temporal preparation, temporal probability, rhythm, aging

## Abstract

Temporal preparation has been consistently shown to be driven by regular rhythms, which are commonly considered to automatically attract attentional resources to on-beat moments, facilitating behavioral performance relative to off-beat moments in both younger and older adults. However, when targets occur more often at off-beat moments such that the rhythm becomes task-disadvantageous, it remains unclear whether older adults can adjust preparatory resources away from on-beat moments and toward the high-probability time point. To address this issue, younger and older adults completed a temporal preparation task at fast (800 ms) and slow (2000 ms) tempos under three conditions: attend-on-beat (rhythmic sequence; 80% on-beat targets), attend-off-beat (rhythmic sequence; 80% off-beat targets), and random (nonrhythmic sequence; 50% each). The results showed that, relative to the random condition, both age groups responded faster at the instructed high-probability time point in both rhythmic conditions, even when it fell off-beat, indicating that temporal probabilities can guide temporal preparation away from a task-disadvantageous on-beat moment toward the task-relevant time point. Moreover, this pattern was observed under both the fast and slow tempos. Together, these findings suggest that older adults preserve the ability to use temporal probabilities to reduce rhythmic distraction across sub-second and supra-second time scales.

## 1. Introduction

Temporal preparation is a critical ability for daily life and refers to the allocation of attentional resources to an expected moment in time, thereby enhancing cognitive processing and optimizing behavioral performance when a relevant stimulus occurs (Coull & Nobre, 1998; Nobre & Van Ede, 2018). Empirical evidence indicates that temporal preparation can be elicited by isochronous stimulus sequences such as regular rhythms, which are common in the natural environment (M. R. Jones et al., 2002; Rohenkohl et al., 2012; Rohenkohl & Nobre, 2011; Sanabria et al., 2011; Sanabria & Correa, 2013). For example, the rhythms of breathing, heartbeat, and the beat in music can all provide predictable temporal structure. Importantly, both younger and older adults can use regular rhythms to enhance temporal preparation, facilitating the processing of events occurring at the predicted beat (Cravo et al., 2013; A. Jones, 2015; Lange, 2009, 2010; Morillon et al., 2016; Ren et al., 2019; Xu et al., 2021a, 2024, 2025).

Rhythm-based temporal preparation is typically considered a largely stimulus-driven and relatively automatic process (Cutanda et al., 2015; De la Rosa et al., 2012; Xu et al., 2021b) and is commonly discussed within the framework of Dynamic Attending Theory (Barnes & Jones, 2000; M. R. Jones, 1976; Large & Jones, 1999). According to this account, when individuals are exposed to a non-random temporal structure with a fixed interval, internal attentional oscillations (i.e., attending rhythms) can spontaneously synchronize with the external temporal pattern, such that attention tends to peak at expected time points. Consequently, events that occur in phase with the rhythm, namely on-beat, are typically processed more efficiently than events occurring off-beat (i.e., earlier or later than the expected time point).

More recently, age-related changes in temporal preparation have been increasingly examined (Capizzi et al., 2022; Gallego Hiroyasu & Yotsumoto, 2020; Shalev et al., 2024; Visalli et al., 2025). Our previous studies also found that the ability to enhance temporal preparation from both auditory and visual isochronous rhythms can be preserved in normal aging (Xu et al., 2024, 2025). In addition, temporal preparation in older adults can be driven not only by rhythms but also by temporal probabilities, implemented by assigning a higher probability of target occurrence to one time point than to alternative time points, resulting in faster responses when targets occur at the high-probability time point, for example, 75% in Chauvin et al. (2016), and 80% in Heideman et al. (2018).

However, research on temporal preparation in aging has primarily focused on a single source of temporal information, such as rhythm or temporal probabilities. Evidence from selective-listening research suggests that, when multiple candidates are available, attention can influence which auditory stream is preferentially tracked and, consequently, which temporal structure is more likely to guide behavior (Ding & Simon, 2012; Kerlin et al., 2010; McAuley et al., 2020; Mesgarani & Chang, 2012). Although these studies focused on competing speech streams rather than on competition between different temporal moments within a single rhythmic context, they suggest that temporal preparation may be shaped not only by rhythmic regularity itself but also by the allocation of task-relevant attention. Thus, when the task-relevant off-beat time point conflicts with the more rhythmically salient on-beat moment, whether older adults can flexibly shift preparation toward the off-beat time point warrants further investigation.

Although previous research has shown that, when rhythmic and probabilistic temporal information is concurrently available and in conflict, younger adults can shift temporal preparation from the on-beat moment to off-beat target time points with a higher probability of occurrence (Breska & Deouell, 2016), it remains unclear whether such flexibility is preserved in older adults. Accumulating evidence suggests that normal aging is associated with changes in temporal processing performance, and that these age-related differences may reflect not only alterations in timing mechanisms per se, but also age-related changes in attentional, working memory, and executive control processes (Baudouin et al., 2006; Lamotte & Droit-Volet, 2017; Mioni et al., 2020; Nowak et al., 2016; Turgeon et al., 2016). Consistent with this view, converging neurobiological evidence indicates that several neural systems implicated in temporal processing, such as cortico-striatal circuits, cerebellar systems, and thalamo-cortical pathways, show age-related changes in structure, function, or connectivity (Bernard & Seidler, 2014; Bo et al., 2014; Goldstone et al., 2018; Petter et al., 2016). Moreover, it has been proposed that age-related differences in temporal processing tend to become more pronounced under conditions of greater task complexity and increased demands on attention and working memory (Baudouin et al., 2006; Mioni et al., 2020). Consequently, when rhythmic temporal information that relies more on relatively automatic processing and probabilistic temporal information that may depend more on attentional and working memory resources (Capizzi et al., 2012, 2013) co-occur and potentially compete, how older adults integrate and weigh these sources remains an open question. Specifically, when the high-probability target time point deviates from the beat, preparatory resources automatically allocated to beat-based moments may no longer be beneficial for target processing. It therefore remains to be determined whether older adults can flexibly shift temporal preparation away from the on-beat moment and toward the high-probability off-beat time point, thereby reducing rhythmic interference. This ability to disengage from a distracting rhythm is also important in everyday contexts; for example, during driving, the rhythmic cadence of windshield wipers may entrain temporal preparation, yet critical hazards (e.g., changes in traffic signals) are not synchronized with this rhythmic timing. Failure to overcome such rhythmic entrainment may increase safety risks.

To address these issues, the present study employed a task-disadvantageous rhythmic context to examine whether older adults can flexibly shift temporal preparation away from on-beat times toward the task-relevant, high-probability target time point when the rhythm is distracting. We utilized a rhythmic temporal preparation task adapted from Breska and Deouell (2016), in which an isochronous or random tone sequence preceded an auditory target; target-timing probabilities were manipulated across three conditions (attend-on-beat, attend-off-beat, and random), and two tempos were tested (fast: 800 ms; slow: 2000 ms). This approach allowed us to assess probability-guided adjustments of temporal preparation under a distracting rhythm across sub-second and supra-second time scales.

## 2. Materials and Methods

### 2.1. Participants

G*Power 3.1.9.7 (Faul et al., 2007) was used to perform an a priori power analysis for a repeated-measures analysis of variance (ANOVA), within–between interaction. An effect size of 0.2 was adopted, with alpha set at 0.05 and power set at 0.8. The analysis indicated that a minimum of 10 participants was required for each age group. According to the convention commonly adopted in ageing research by the World Health Organization (WHO), individuals aged 60 years and above are generally classified as older adults (World Health Organization & Edwards, 2002). Consequently, our study recruited 24 younger adults (14 females; 18–21 years, M = 19.42, SD = 1.10) and 24 older adults (14 females; 66–79 years, M = 72.04, SD = 3.65), ensuring substantial statistical power for achieving the primary goal of our research.

To obtain the two age groups, we recruited younger adults from Ningbo University of Technology and older adults from the surrounding community. According to self-report, all participants were right-handed, had normal or corrected-to-normal vision, and reported normal hearing. No participant had a history of psychiatric or neurological disorders or was taking psychotropic or vasoactive medication. Additionally, none of the participants had received professional music training or had played any musical instrument during the three years preceding the study. The research procedures were reviewed and approved by the institutional ethics committee, and all participants signed written informed consent prior to participation. To screen for dementia, older adults underwent the Mini-Mental State Examination (Folstein et al., 1975) to exclude individuals with dementia. All the older participants achieved the threshold score of 27 (M = 29.25, SD = 0.79).

### 2.2. Apparatus and Stimuli

E-Prime 3.0 (Schneider et al., 2002) was used to present the stimuli and record participants’ reaction times (RTs). Visual stimuli were displayed at the center of a 27-inch monitor (1920 × 1080 pixels; 60 Hz) on a black background, with a viewing distance of 60 cm from the screen center. Auditory stimuli were delivered through two loudspeakers positioned 50 cm from the participant, located at the left-front and right-front positions, at a comfortable listening level.

**Figure 1:**
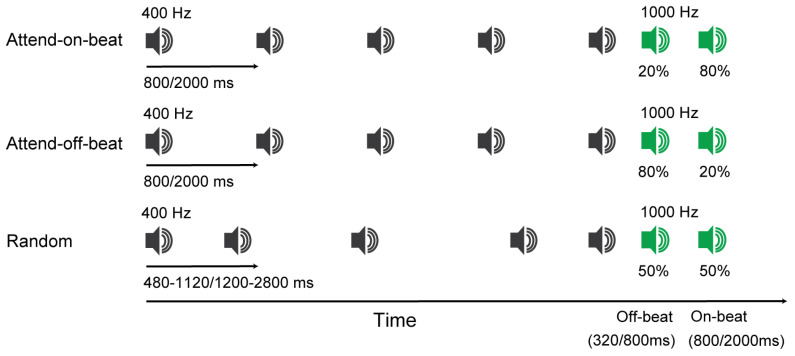
Schematic representation of events in a trial across conditions. A 400–Hz tone sequence was presented at a fast (800 ms) or slow (2000 ms) tempo, followed by a 1000–Hz target tone occurring at either the on-beat or off-beat SOA. Percentages shown beneath the target stimuli represent the likelihood that the target occurred at the indicated time point in each condition.

Fixation was indicated by a centrally presented white “+” symbol subtending 0.7° × 0.7° of visual angle. The auditory stimulus sequence consisted of four or five consecutive 400 Hz pure tones, each lasting 100 ms, and followed either an isochronous (fixed-interval) or random-interval timing pattern. For the isochronous sequence, tones occurred at a constant inter-onset interval (IOI), with 800 ms for the fast tempo and 2000 ms for the slow tempo. For the random sequence, the IOIs varied across tones and were drawn from 480, 640, 800, 960, or 1120 ms (fast tempo) or 1200, 1600, 2000, 2400, or 2800 ms (slow tempo), with the order of these intervals randomized within each trial. The target was a 1000 Hz pure tone, presented for 100 ms (see Figure 1).

### 2.3. Procedure

Each trial began with a 400–Hz tone sequence, presented in either an isochronous or random pattern. Participants were instructed to respond to the 1000–Hz target tone by clicking the left mouse button with the right index finger as rapidly as possible, while avoiding responding before target onset. The fixation remained at the center of the screen throughout the trial and disappeared immediately after the response. Responses were allowed within a maximum time window of 1200 ms. A 500 ms inter-trial interval (ITI) followed each trial, during which the screen was blank.

Participants received task instructions in both written and spoken formats. Prior to each experimental block, participants were notified of the forthcoming block condition (attend-on-beat, attend-off-beat, or random) and were encouraged to use the target-timing probabilities associated with the condition to prepare for target onset. For the attend-on-beat condition, the target occurred at the on-beat target stimulus onset asynchrony (SOA; interval between the onset of the last 400–Hz tone and the onset of the target tone) in 80% of trials and at the off-beat target SOA in the remaining 20%. The on-beat target SOA matched the rhythmic IOI (fast: 800 ms; slow: 2000 ms), whereas the off-beat SOA was shorter (fast: 320 ms; slow: 800 ms). For the attend-off-beat condition, these proportions were reversed (20% on-beat and 80% off-beat). For the random condition, the target occurred at the on-beat and off-beat SOAs with equal probability (50% at each time point).

### 2.4. Design

The experiment employed a mixed factorial design with Age group (younger, older) as a between-participants factor and three within-participants factors: Condition (attend-on-beat, attend-off-beat, random), Tempo (fast, slow), and Target SOA (on-beat, off-beat). Each participant completed 12 experimental blocks, including 6 fast-tempo blocks and 6 slow-tempo blocks, with 40 trials per block. Across participants, the presentation order of the tempo blocks was counterbalanced. Within each tempo, the order of the three condition blocks (random, attend-on-beat, attend-off-beat) was randomized. Before each condition block, participants completed a 10-trial practice block.

### 2.5. Data Analysis

RT was measured from the onset of the target stimulus to the participant’s response. Practice trials were not included in the analysis. Additionally, trials were excluded if responses occurred before target onset (anticipatory responses), if no response was made following target onset (omission errors), or if RTs exceeded three standard deviations from each participant’s mean RT for each dependent measure. The proportion of trials removed as outliers was similar across age groups (younger: 1.82%; older: 1.48%). No statistically significant group difference was found in the independent-samples *t*-test, t(46) = 0.67, *p* = 0.504.

Mean RTs for correct responses were analyzed using a four-way mixed-design ANOVA with Age group as a between-participants factor and Condition, Tempo, and Target SOA as within-participants factors (see Design for factor levels). Before conducting the ANOVA, the distribution of RTs was assessed by examining skewness and kurtosis. All skewness and kurtosis values fell within acceptable limits (absolute skewness values < 1; absolute kurtosis values < 2), indicating that the RT distributions could be regarded as sufficiently normal for parametric analyses (Kim, 2013; West et al., 1995). To examine the costs and benefits of attentional shifting beyond the Target SOA effect (Niemi & Näätänen, 1981), we performed planned contrasts comparing attend-on-beat vs. random and attend-off-beat vs. random, separately for each Target SOA (on-beat and off-beat). When the sphericity assumption was violated, Greenhouse–Geisser-corrected *p* values were reported together with the uncorrected degrees of freedom. Results were considered statistically significant at *p* < 0.05, and effect sizes were quantified using partial eta squared (*η_p_*^2^). All statistical analyses were conducted in IBM SPSS Statistics 19.0 (IBM Corp., Armonk, NY, USA).

## 3. Results

### 3.1. Overall Analyses

Table 1 presents the mean RTs for all conditions. The four-way mixed-design ANOVA revealed a significant main effect of Age group [F(1, 46) = 60.475, *p* < 0.001, *η_p_*^2^ = 0.568], with younger participants showing faster RTs (251.25 ms) than older participants (295.53 ms). A significant main effect of Tempo was also observed [F(1, 46) = 90.22, *p* < 0.001, *η_p_*^2^ = 0.662]; mean RTs were shorter for the fast tempo (263.28 ms) relative to the slow tempo (283.50 ms). In addition, significant main effects were found for Condition [F(2, 92) = 14.436, *p* < 0.001, *η_p_*^2^ = 0.239] and Target SOA [F(1, 46) = 48.933, *p* < 0.001, *η_p_*^2^ = 0.515]. Significant interactions were observed between Condition and Age group [F(2, 92) = 4.602, *p* = 0.013, *η_p_*^2^ = 0.091], and between Condition and Target SOA [F(2, 92) = 130.514, *p* < 0.001, *η_p_*^2^ = 0.739]. Moreover, the three-way interaction among Tempo, Condition, and Target SOA was significant [F(2, 92) = 3.133, *p* = 0.049, *η_p_*^2^ = 0.064]. Given the significant Condition × Age group interaction, separate three-way repeated-measures ANOVAs were run for each age group, with Condition, Tempo, and Target SOA entered as within-subject factors.

### 3.2. Younger Adults

For the younger group, significant main effects were found for Tempo [F(1, 23) = 72.157, *p* < 0.001, *η_p_*^2^ = 0.758], Condition [F(2, 46) = 5.402, *p* = 0.008, *η_p_*^2^ = 0.190], and Target SOA [F(1, 23) = 18.680, *p* < 0.001, *η_p_*^2^ = 0.448]. The interaction between Tempo and Target SOA also reached significance [F(1, 23) = 6.672, *p* = 0.017, *η_p_*^2^ = 0.225]. As expected, the analysis revealed a significant interaction between Condition and Target SOA [F(2, 46) = 78.827, *p* < 0.001, *η_p_*^2^ = 0.774], as well as a significant three-way interaction among Tempo, Condition, and Target SOA [F(2, 46) = 5.818, *p* = 0.008, *η_p_*^2^ = 0.202], indicating that temporal preparation differed across conditions as a function of tempo. To assess resource allocation in each condition, we conducted two planned interaction contrasts, in which each rhythmic condition was compared with the random condition separately at the fast and slow tempos, followed by paired-samples *t*-tests within each Target SOA.

**Figure 2:**
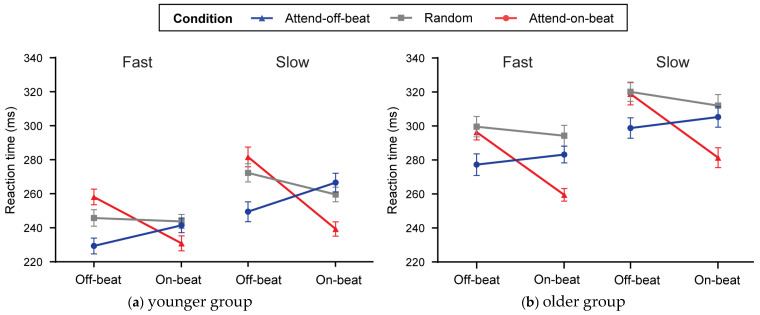
Mean RT (ms) as a function of Tempo (fast, slow), Target SOA (off-beat, on-beat), and Condition (attend-on-beat, random, attend-off-beat), shown separately for the younger (**a**) and older (**b**) groups. Error bars represent the standard error of the mean.

**Figure 3:**
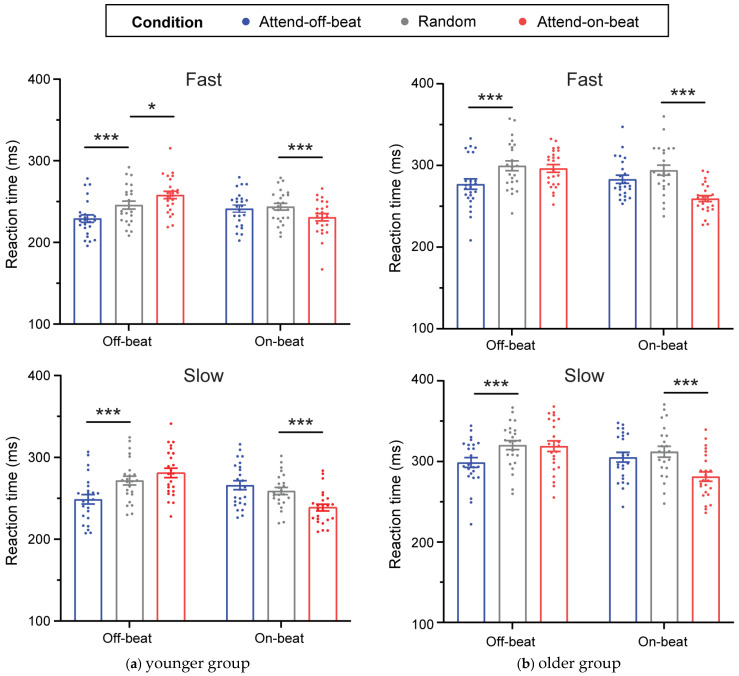
Actual plots for mean reaction times (RTs) as a function of Tempo (fast, slow), Target SOA (on-beat, off-beat), and Condition (attend-on-beat, attend-off-beat, random), shown separately for the younger (**a**) and older (**b**) groups. Error bars reflect the standard error of the mean. Statistical significance is indicated by *** *p* < 0.001, * *p* < 0.05.

In the fast tempo condition, a significant interaction effect was observed when comparing the attend-on-beat and random conditions [F(1, 23) = 31.696, *p* < 0.001, *η_p_*^2^ = 0.579]. Follow-up tests showed that responses to off-beat targets were slower in the attend-on-beat condition than in the random condition (t(23) = 2.97, *p* = 0.007, Cohen’s d = 0.61), whereas responses to on-beat targets were faster (t(23) = −4.39, *p* < 0.001, Cohen’s d = 0.90). A significant interaction effect was also observed when comparing the attend-off-beat and random conditions [F(1, 23) = 12.362, *p* = 0.002, *η_p_*^2^ = 0.35]. Follow-up tests showed that responses to off-beat targets were faster in the attend-off-beat condition than in the random condition (t(23) = −5.14, *p* < 0.001, Cohen’s d = 1.05), whereas responses to on-beat targets did not differ between the two conditions (t(23) = −0.58, *p* = 0.570) (Figure 2a and Figure 3a, Fast).

In the slow tempo condition, a significant interaction effect was observed when comparing the attend-on-beat and random conditions [F(1, 23) = 27.07, *p* < 0.001, *η_p_*^2^ = 0.541]. Follow-up tests showed that responses to off-beat targets did not differ between the attend-on-beat and random conditions (t(23) = 1.64, *p* = 0.115), whereas responses to on-beat targets were faster in the attend-on-beat condition (t(23) = −8.45, *p* < 0.001, Cohen’s d = 1.72). A significant interaction effect was also observed when comparing the attend-off-beat and random conditions [F(1, 23) = 47.163, *p* < 0.001, *η_p_*^2^ = 0.672]. Follow-up tests showed that responses to off-beat targets were faster in the attend-off-beat condition than in the random condition (t(23) = −5.34, *p* < 0.001, Cohen’s d = 1.09), whereas responses to on-beat targets did not differ between the two conditions (t(23) = 1.67, *p* = 0.109) (Figure 2a and Figure 3a, Slow).

### 3.3. Older Adults

For the older group, significant main effects were found for Tempo [F(1, 23) = 34.299, *p* < 0.001, *η_p_*^2^ = 0.599], Condition [F(2, 46) = 11.37, *p* < 0.001, *η_p_*^2^ = 0.331], and Target SOA [F(1, 23) = 30.642, *p* < 0.001, *η_p_*^2^ = 0.571]. As expected, the analysis revealed a significant interaction between Condition and Target SOA [F(2, 46) = 54.790, *p* < 0.001, *η_p_*^2^ = 0.704], indicating that temporal preparation differed across conditions. Following the same analyses as in the younger group, we performed the planned interaction contrasts separately for the fast and slow tempos, followed by paired-samples *t*-tests within each Target SOA.

In the fast tempo condition, a significant interaction effect was observed when comparing the attend-on-beat and random conditions [F(1, 23) = 38.024, *p* < 0.001, *η_p_*^2^ = 0.623]. Follow-up tests showed that responses to on-beat targets were faster in the attend-on-beat condition than in the random condition (t(23) = −5.98, *p* < 0.001, Cohen’s d = 1.220), whereas responses to off-beat targets did not differ between the two conditions (t(23) = −0.54, *p* = 0.596). A significant interaction effect was also observed when comparing the attend-off-beat and random conditions [F(1, 23) = 4.759, *p* = 0.04, *η_p_*^2^ = 0.171]. Follow-up tests showed that responses to off-beat targets were faster in the attend-off-beat condition than in the random condition (t(23) = −3.84, *p* < 0.001, Cohen’s d = 0.785), whereas responses to on-beat targets did not differ between the two conditions (t(23) = −1.59, *p* = 0.126) (Figure 2b and Figure 3b, Fast).

In the slow tempo condition, a significant interaction effect was observed when comparing the attend-on-beat and random conditions [F(1, 23) = 24.849, *p* < 0.001, *η_p_*^2^ = 0.519]. Follow-up tests showed that responses to on-beat targets were faster in the attend-on-beat condition than in the random condition (t(23) = −6.74, *p* < 0.001, Cohen’s d = 1.376), whereas responses to off-beat targets did not differ between the two conditions (t(23) = −0.21, *p* = 0.837). A significant interaction effect was also observed when comparing the attend-off-beat and random conditions [F(1, 23) = 6.215, *p* = 0.02, *η_p_*^2^ = 0.213]. Follow-up tests showed that responses to off-beat targets were faster in the attend-off-beat condition than in the random condition (t(23) = −5.61, *p* < 0.001, Cohen’s d = 1.146), whereas responses to on-beat targets did not differ between the two conditions (t(23) = −1.23, *p* = 0.230) (Figure 2b and Figure 3b, Slow).

## 4. Discussion

Across fast and slow tempos, in both younger and older adults, condition significantly interacted with target SOA when the rhythmic conditions (attend-on-beat and attend-off-beat) were compared with the random condition (see Figure 2). This pattern is consistent with Breska and Deouell (2016), who reported a comparable functional effect in younger adults. Importantly, however, their study used a visual rhythmic sequence, raising the question of whether the ability to shift preparation away from on-beat moments is limited to the visual modality. This concern is particularly relevant given the commonly assumed advantage of the auditory modality for temporal processing: attention tends to be more strongly entrained by auditory rhythms, making it more difficult to disengage preparation from on-beat moments in an auditory rhythmic context than in a visual rhythmic context (Ortega et al., 2014; Penney et al., 2000; Recanzone, 2003; Repp & Penel, 2002, 2004). Addressing this issue directly, the present study used an auditory rhythmic paradigm and observed the same functional pattern, further extending it to older adults. Accordingly, our findings suggest that the ability to shift temporal preparation toward the task-relevant, high-probability time point when the rhythm is task-disadvantageous is not confined to the visual modality and may not be simply determined by modality-based timing advantages; rather, it appears to reflect a strategic adjustment of temporal preparation that can be expressed across modalities and age groups.

Despite the overall correspondence of the interaction pattern with that reported by Breska and Deouell (2016), the present findings differed in how the effect was expressed in the critical contrast for assessing the ability to disengage from on-beat moments and shift preparation to the high-probability off-beat time point (attend-off-beat vs. random). In their study, when off-beat was the high-probability time point, shifting temporal preparation away from the on-beat moment was expressed mainly as a cost at the non-instructed on-beat time point, without a corresponding benefit at the off-beat time point; by contrast, in our attend-off-beat condition, the off-beat time point showed a clear RT benefit, with faster responses to off-beat targets than in the random condition (see Figure 3). One possible account concerns the degree of temporal dispersion in the random condition. Unlike Breska and Deouell (2016), who implemented the random condition as a narrow jitter around a fixed IOI (fast: 650–1050 ms; slow: 1200–1600 ms), the present study used a more dispersed range of IOIs in the random sequence (fast: 480–1120 ms; slow: 1200–2800 ms), which may introduce greater temporal uncertainty—especially in the slow tempo—and thereby weaken baseline preparation and slow overall responding in the random condition (Bekius et al., 2016; Brochard et al., 1999; Horr & Di Luca, 2015; Madison & Merker, 2002). This possibility is consistent with our data in that mean RTs in the random condition were overall slower in the slow than in the fast tempo. Against this background, relative to a slower random baseline, probability-guided preparation may be more likely to be expressed as an RT benefit at the instructed high-probability time point, making benefits more prominent than costs in comparisons with the random condition. It should also be noted that, in the present design, the interval preceding the off-beat time point was always shorter than that preceding the on-beat time point. Previous work has suggested that longer foreperiods are associated with greater temporal uncertainty and less precise temporal prediction, which may in turn slow responding (Gibbon et al., 1984; Müller-Gethmann et al., 2003). From this perspective, the shorter interval preceding the off-beat time point may have supported more precise temporal preparation, thereby facilitating, at least in part, the shift in preparation from the on-beat moment to the high-probability off-beat time point. This possibility, however, remains to be tested more directly in future studies.

In the present study, older adults reliably used temporal probabilities to form temporal preparation, even when the high-probability time point deviated from the rhythmically expected on-beat moment. This finding bears on a long-standing debate about whether temporal orienting based on probabilistic timing information is preserved in healthy aging. Zanto et al. (2011) reported that older adults failed to show such behavioral benefits from the probabilistic temporal information conveyed by visual cues, whereas Chauvin et al. (2016) observed robust temporal-orienting benefits in older adults when target timing was predicted by auditory cues (75% validity) (Chauvin et al., 2016). Our results are more consistent with the blocked manipulation reported by Chauvin et al. (2016), in which auditory cues conveyed temporal predictions and the cue–time probability mapping remained stable within blocks; by contrast, Zanto et al. (2011) used trial-by-trial visual cues. Given the commonly assumed advantage of audition for temporal processing (Ortega et al., 2014; Penney et al., 2000; Recanzone, 2003), and the fact that manipulating temporal preparation between blocks tends to produce more robust temporal-orienting effects than trial-by-trial manipulations (Correa et al., 2004, 2006; Trivino et al., 2010), both cue modality and the stability of the cue–time mapping may influence older adults’ ability to translate temporal probabilities into behavioral benefits. Further work that orthogonally manipulates these factors will be required to clarify how each shape probability-based temporal orienting in aging.

It is noteworthy that older adults were overall slower than younger adults in the present study, a pattern consistent with the general slowing commonly observed in normal aging (Eckert et al., 2010; Hardwick et al., 2022; Salthouse, 1996, 2000). Such slowing may reflect broader age-related reductions in processing speed and, potentially, decreased efficiency in certain aspects of cognitive control (Dexter & Ossmy, 2023; Sweeney et al., 2001). Importantly, however, despite this overall slowing, older adults were still able to use temporal probability information to guide preparation, even when the high-probability time point conflicted with the rhythmically salient on-beat moment. This preserved ability may reflect compensatory processes in healthy aging. As suggested in previous accounts of cognitive aging and time perception, older adults may compensate for age-related decline by recruiting additional cognitive resources, including attention and working memory, relying more strongly on environmental support, and possibly engaging timing-related neural circuits beyond the age-sensitive fronto-striatal timing network (Lindenberger & Mayr, 2014; Reuter-Lorenz & Cappell, 2008; Turgeon et al., 2016). Within this framework, the present findings are consistent with the possibility that probability-based temporal preparation may remain relatively preserved in older adults under task conditions that provide sufficient external support and do not place excessive demands on compensatory processes; by contrast, age-related deficits may become more apparent when task demands place greater strain on attention, working memory, and other compensatory resources (Reuter-Lorenz & Cappell, 2008). However, as the present study provides behavioral evidence only, this compensatory account remains tentative and should be further examined using neurophysiological or neuroimaging methods. It should also be noted that the older group in the present study covered a relatively broad age range (66–79 years). Although these participants did not fall within the age range more commonly regarded in the cognitive aging literature as representing a more advanced stage of old age, typically 80 years and above (or, in some studies, 85 years and above), potential age heterogeneity within the older group cannot be entirely ruled out (Baltes & Smith, 2003; Calero et al., 2013; Merenstein & Bennett, 2022; Navarro & Calero, 2018; Rojas et al., 2023). Future studies with larger samples should further examine whether probability-based temporal preparation differs across more fine-grained subgroups of older adults.

## 5. Conclusions

In conclusion, in the attend-on-beat condition (80% on-beat targets), both younger and older adults responded faster to on-beat targets than in the random condition; critically, in the attend-off-beat condition (80% off-beat targets), both groups responded faster to off-beat targets than in the random condition. This pattern indicates that, even when the rhythmically entrained on-beat moment is task-disadvantageous, both groups can use temporal probabilities to flexibly prioritize temporal preparation at the task-relevant, high-probability time point. Moreover, this functional pattern was observed under both the fast (800 ms) and slow (2000 ms) tempos. Together, these findings suggest that rhythmic entrainment is not strictly obligatory but can be modulated by probability-based preparation, such that the ability to use temporal probabilities to reduce rhythmic interference appears to be preserved in healthy aging across sub-second and supra-second time scales. These findings offer insight into how multiple sources of temporal information may be integrated in healthy aging to support adaptive behavior in dynamic environments.

## Figures and Tables

**Table 1:** Mean RTs (ms) for each age group (younger, older), tempo (fast, slow), condition (attend-on-beat, attend-off-beat, random), and target SOA (off-beat, on-beat).

	Younger Group				Older Group			
	Fast		Slow		Fast		Slow	
	Off-Beat	On-Beat	Off-Beat	On-Beat	Off-Beat	On-Beat	Off-Beat	On-Beat
Attend-on-beat	258 (5)	231 (4)	281 (6)	239 (4)	296 (5)	259 (4)	319 (7)	281 (6)
Attend-off-beat	229 (5)	241 (4)	249 (6)	266 (6)	277 (6)	283 (5)	299 (6)	305 (6)
Random	246 (5)	244 (4)	272 (5)	259 (4)	300 (6)	294 (6)	320 (6)	312 (6)

Values in parentheses are standard errors of the mean.

## Data Availability

The datasets generated and analyzed during the current study are available from the corresponding author on reasonable request.
